# Long-term fate of the incised urethral plate in Snodgrass procedure; A real concern does exist

**DOI:** 10.1016/j.eucr.2020.101216

**Published:** 2020-04-23

**Authors:** Tariq O. Abbas, Adrian Charles, Mansour Ali, Joao Luiz Pippi Salle

**Affiliations:** a Division of Pediatric Urology, Departement of Surgery, Sidra Medicine, Doha, Qatar; b College of Medicine, Qatar University, Doha, Qatar; c Weill Cornel Medical College-Qatar, Doha, Qatar; d Regenerative Medicine Research Group, Dapartment of Health Science and Technology, Aalborg Univerisity, Aalborg, Denmark; e Pathology Department, Sidra Medicine, Doha, Qatar; f Department of Surgery, Sidra Medicine, Doha, Qatar

**Keywords:** Hypospadias, Urethra, Histology, Acquired chordee, Fibrosis, Incised plate

## Abstract

We present here a case of a patient post tabularized incised plate urethroplasty for distal hypospadias without chordee who developed urethral stenosis and acquired curvature along the territory of the incised plate necessitating a redo surgery. The histological analysis of the incised urethral plate revealed absence of smooth muscles, vessels and elastin fibers within the area of the incised plate which could explain the poor compliance of this segment and the development of the curvature. To our knowledge, this case is the first in humans displaying the long-term histological findings of healing post tabularized incised plate urethroplasty.

## Introduction

Hypospadias is a common urological congenital malformation that occurs an approximate incidence of 38 per 10,000 males. Although more than 300 distinct procedures for hypospadias reconstruction exist, no one is considered the ideal technique. A successful result depends mainly on the surgeon's skills, availability of sufficient tissue for urethral repair and judgment of the best fitting procedure in an individual case.

Snodgrass, in 1998, popularized to incise and tubularize the urethral plate (TIP), relying on re-epithelization and granulation of the dorsal urethra. That technique has been globally well accepted despite the lack of clear understanding of the healing of the incised plate. This could be a reason why some cases of narrow and inelastic plates healed inappropriately.

Here in, we present the histological analysis of a case post TIP who developed severe fibrosis of the incised urethral plate and acquired curvature necessitating a redo surgical intervention.

### Case report

**Fig. 1:**
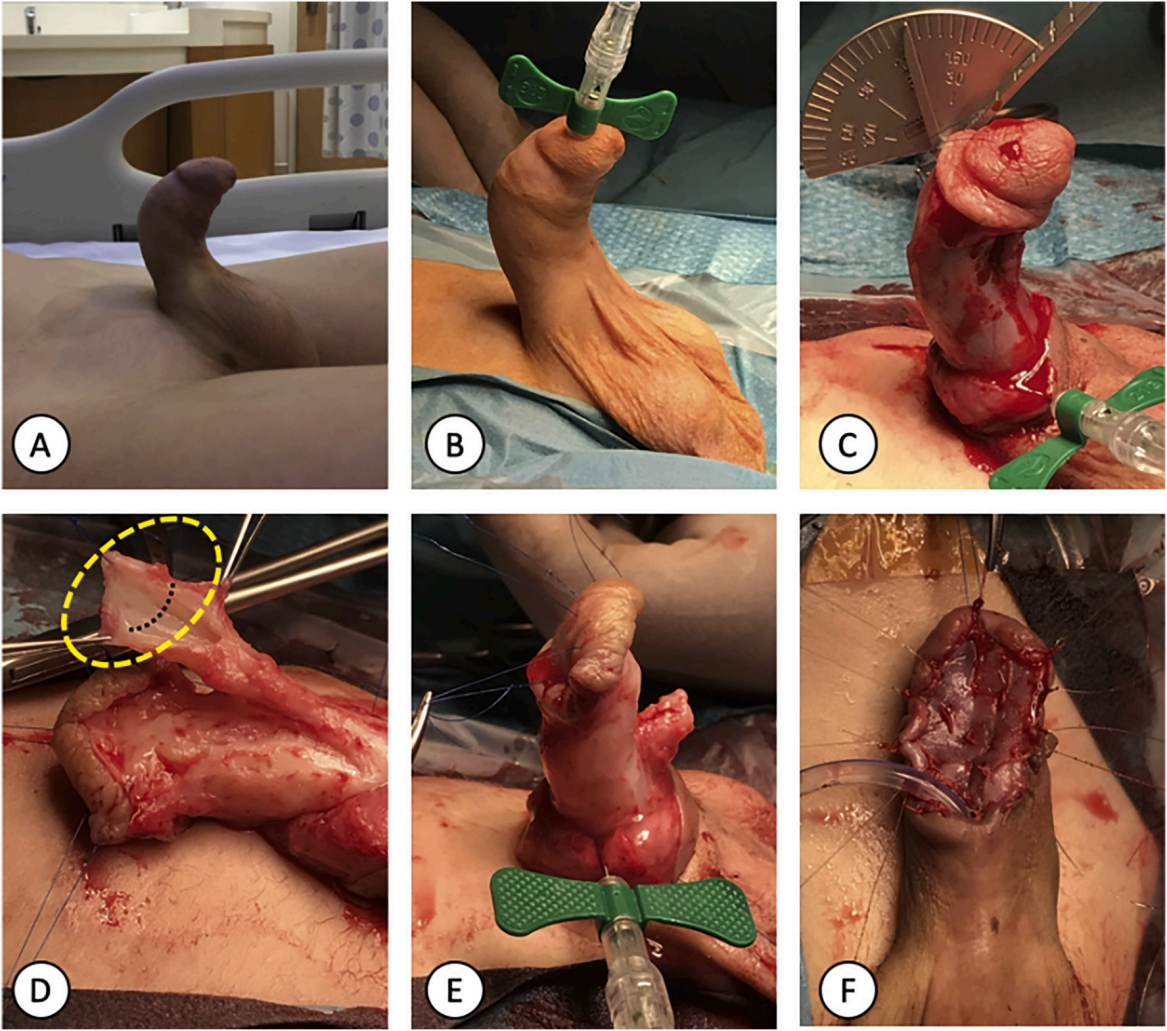
A. The patient has a significant ventral curvature noticed on spontaneous erection. B. Intraoperatively and before degloving, artificial erection test revealed the same degree of ventral chordee. C. Repeat erection test following skin degloving unfolds the same level of midshaft (60°) ventral penile curvature. D. Excision of the urethral plate was done, and the yellow dashed area was sent to histopathology (dashed black line represents the sectioning orientation). E. Erection test repeated after excision of the urethral plate demonstrating a straight penis. F. Buccal mucosa graft was fixed to stage the repair of the hypospadias. (For interpretation of the references to colour in this figure legend, the reader is referred to the Web version of this article.)

**Fig. 2:**
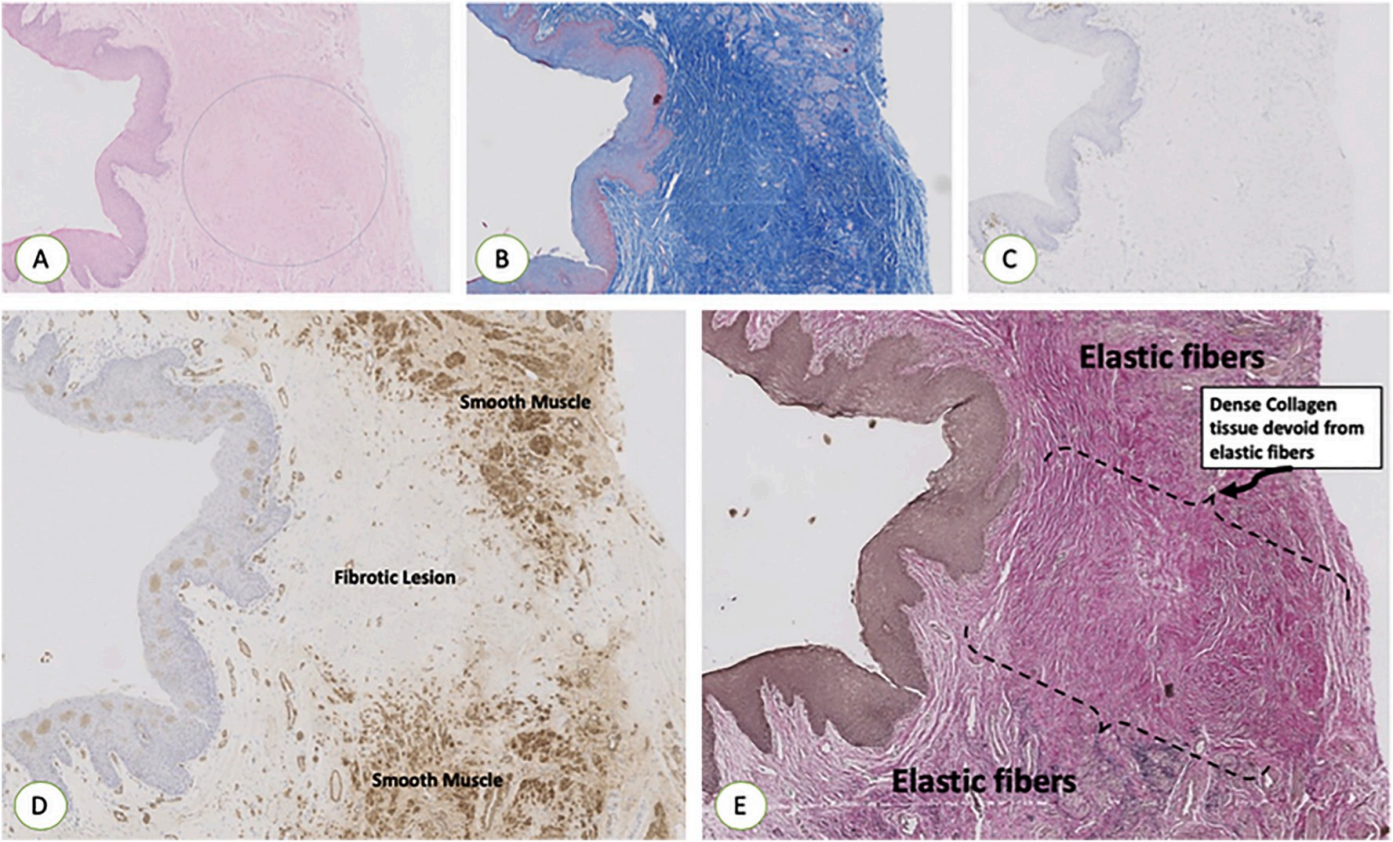
A. H&E stain is showing the healing of the incised area with no regrowth of smooth muscles or vessels. B. MTC stain showing the healing of the incised area with no regrowth of smooth muscles but dense collagen tissue **(Blue).** C. IHC using CD3 T cells showing the absence of any inflammatory reaction in the incised area. D. IHC using SMA is showing the healing of the incised area with no regrowth of smooth muscles. E. Van Gieson stain showing the healing of the incised area with no regrowth of elastic fibers. (H&E: hematoxylin and Eosin, MTC: Masson Trichrome, IHC: Immunohistochemistry, SMA: Smooth Muscle Actin). (For interpretation of the references to colour in this figure legend, the reader is referred to the Web version of this article.)

A 7-year-old male with thrombocytopenia and hypospadias with intact prepuce (discovered during ritual circumcision). He underwent TIP repair for the hypospadias, glans width was 12 mm. The immediate postoperative period was uneventful. However, he developed significant stenosis and required several dilations and progressive acquired ventral curvature. A redo staged buccal urethroplasty was eventually performed 7 years later. During the redo surgery, the erection test showed ventral (60°) curvature (Fig. 1) that persisted after degloving and mandated excision of the distal urethra. The excised urethral plate was sent to histopathology (Fig. 2). Buccal mucosa graft was utilized to stage the repair of his hypospadias.

## Discussion

Our case clearly documents the potential for significant complications post TIP repair such as fistula, stenosis and ventral curvatures.

The healing process of an incised dorsal urethra is unclear. Bleustein et al.^1^ examined the acute healing of the incised plate urethroplasty in dogs that showed the presence of normal connective tissue and vessels. However, Holland and Smith clinically observed no evidence that incising the urethral plate increased the final urethral diameter. In their article, shallow urethral plate (<8 mm width) were more likely to have more complications.^2^

Several authors support the use of TIP repair reporting improved flow rates overtime which would attest for the good outcomes in many patients. However, a small percentage of patients develop significant complications.^3^

Urine is believed to be cytotoxic to the regrowth of tissues in the incised urethral plate area and rich furnished in protamine sulfate, products of low molecular weight and cationic materials that are mainly accountable for its nonselective and great cytotoxicity.^4^ In an effort to improve healing of the neourethra following the TIP urethroplasty, grafting of the dorsal incised (GTIP) region applying the inner foreskin has been reported by different authors.^5^

To our knowledge, this case introduces the first detailed histological analysis in humans of the incised urethral plate exploring the long-term nature of healing post tabularized incised plate urethroplasty.

## Ethics statement

Our study did not require an ethical board approval because no experimental study was conducted on patients beyond normal clinical care pathway. However, an informed consent from the parent of this patient was secured.

## Funding

No funding or grant support.

## Authorship

All authors attest that they meet the current ICMJE criteria for Authorship.

## Declaration of competing interest

All authors of this manuscript have directly participated in planning, execution, and analysis of this study. The contents of this manuscript have not been copyrighted of published previously. The contents of this manuscript are not now under consideration for publication elsewhere. No financial support or incentive has been provided for this manuscript. I am one author signing on behalf of all co-authors of this manuscript, and attesting to the above.
